# Effects of different treatments for obstructive sleep apnea on temporomandibular joint: a randomized clinical trial

**DOI:** 10.1186/s12903-024-04623-w

**Published:** 2024-08-11

**Authors:** Amira A.M.M. Attia, Sally S Awad, Manar Mansour, Hemmat Baz, Khaled M Zahran, Abdelbaset M. Saleh

**Affiliations:** 1 Oral and Maxillofacial Surgery, Faculty of Dentistry, Mansoura University, Mansoura, Egypt; 2 Diagnostic and Interventional Radiology, Faculty of Medicine, Mansoura University, Mansoura, Egypt; 3 Phoniatrics Unit, Faculty of Medicine, Mansoura University, Mansoura, Egypt; 4 Fixed Prosthodontics, Faculty of Dentistry, Mansoura University, Mansoura, Egypt; 5 Chest Medicine, Sleep Disorder Breathing Unit, Faculty of Medicine, Mansoura University, Mansoura, Egypt

**Keywords:** Obstructive sleep apnea, Temporomandibular disorder, Mandibular advancement device, Continuous positive airway pressure, oral myofunctional therapy, Pain

## Abstract

**Background:**

In recent years, obstructive sleep apnea (OSA) has been increasingly recognized as a significant health concern. No previous studies assessed the effect of recommended treatment modalities of patients with OSA on the temporomandibular joint (TMJ). The current study aimed to evaluate the effect of different treatment modalities of OSA, such as continuous positive airway pressure (CPAP), mandibular advancement device (MAD), and oral myofunctional therapy (OMT) on subjective symptoms, clinical, and radiographic signs of temporomandibular disorders.

**Patients & Methods:**

This hospital-based prospective randomized controlled clinical trial study was approved by the institutional review board and formal patient consent, 39 OSA patients, ranging in age from 19 to 56 after confirmation with full night Polysomnography (PSG) with healthy TMJ confirmed clinically and radiographically with magnetic resonance imaging (MRI) were randomly allocated into three treatment groups. Group 1: 13 patients were managed with CPAP after titration, group 2: 13 patients were managed with digitally fabricated MAD, and group 3: 13 patients were managed with OMT. The following parameters were evaluated before and 3 months after the intervention. Pain using a visual analogue scale (VAS), maximum inter-incisal opening (MIO), lateral movements, and clicking sound of TMJ. MRI was done before and 3 months after the intervention.

**Results:**

Out of the 83 patients enrolled, 39 patients completed the treatment. There were no statistically significant differences in lateral jaw movements or clicking, and no significant difference in MRI findings between the three studied groups before and after the intervention. The OMT group showed a statistically significant difference in pain (*p* = 0.001), and MIO (*p* = 0.043) where patients experienced mild pain and slight limitation in mouth opening after 3 months of follow-up in comparison to MAD and CPAP groups.

**Conclusion:**

CPAP and MAD are better for preserving the health of TMJ in the treatment of OSA patients. While OMT showed mild pain and slight limitation of MIO (that is still within the normal range of mouth opening) compared to CPAP and MAD.

**Trial registration:**

The study was listed on www.clinicaltrials.gov with registration number (NCT05510882) on 22/08/2022.

**Supplementary Information:**

The online version contains supplementary material available at 10.1186/s12903-024-04623-w.

## Background

Obstructive sleep apnea is a recurrent upper airway obstruction, that results in oxygen desaturations and awakening from sleep [1]. Five or more episodes of complete (apnea) or incomplete (hypopnea) obstruction per hour during sleep occurring in the existence of sleep-related symptoms is defined as OSA. Its severity is described by the apnea-hypopnea index (AHI). In mild OSA ( AHI ≥ 5 < 15 events/h, moderate (AHI ≥ 15 < 30 events/h), and in severe OSA (AHI ≥ 30 events/h) [2]. About one billion individuals between the ages of 30 and 69 years worldwide have obstructive sleep apnea (OSA) [3]. OSA’s global prevalence is rising because of increased obesity which is the major risk factor for the disease [4].

Polysomnography (PSG) is the “gold standard” for diagnosing OSA and is the most commonly used method [5]. This method records sleep time, the average number of apnoeas and hypopnoeas per hour of sleep, sleep efficiency, and evaluates OSA severity [6, 7]. However, PSG has limitations, as the need for qualified staff, expensive equipment and a sleep monitoring room, and the effect of multiple electrodes on the patient’s sleep [5].

Up to now, many feasible modalities [8] have been offered for the treatment of OSA as; medication, weight loss, and pharyngeal surgery, however, the typical treatment for OSA (moderate to severe) is the use of continuous positive airway pressure (CPAP) overnight [9].

American academy of sleep medicine and American academy of dental sleep medicine clinical practice guideline recommend the use of mandibular advancement devices (MAD) as an effective alternative for those who prefer oral appliances [10–12]. To make a custom acrylic resin MAD, it is important to make impressions, record the amount of advancement and vertical opening, and transfer these records to an articulator [12, 13]. The dimensional stability of the material used in the manufacturing process affects the accuracy of the completed appliance [14]. Conventional impression procedures are uncomfortable to most patients as they may cause a gag response [15].

Currently, using computer-aided design/computer-aided manufacturing (CAD/CAM), 3D scanning, and additive manufacturing technologies may be applied for the fabrication of monobloc, non-adjustable MADs using a digital workflow [16].

On the other hand, in patients with low compliance with CPAP or MAD, OMT is settled as a non-invasive, alternative management for OSA [12].

Patients having OSA signs and symptoms are more likely to present the first onset temporomandibular disorder (TMD), [17] and also has been shown that nearly 90% of these patients have comorbid sleep disorders [18]. Creed [19] described a reciprocal relationship between chronic musculoskeletal pain and sleep. Musculoskeletal pain is characterized by pain or dysfunction involving the TMJ, muscles of mastication, or both [20]. TMDs are a significant problem for the adult population, as they are the second most common musculoskeletal pain source after chronic low-back pain [21]. Many studies indicate that TMD may have a negative effect on patients’ lives, manifested as chronic pain, and loss of energy due to physical or emotional disorders, anxiety/depression [22]. Another studies conducted on TMD patients in Polish population showed that while pain is one component that affects the perceived satisfaction with life, the high prevalence of stress, anxiety and depression in these patients can also influence the level of life satisfaction [23, 24].

Based on the above-mentioned, the current study aimed to assess the subjective symptoms, clinical and radiographic signs of temporomandibular disorders related to the use of CPAP, MAD, and OMT in the treatment of obstructive sleep apnea.

## Patients and methods

### Ethical statement

The study protocol was approved by the institutional review board of the Faculty of Medicine, Mansoura University (NO. R.22.06.1737). The Helsinki Declaration and the guidelines set by the institutional ethics committee were adhered to in all aspects of this study’s activities. Participants in the study provided written informed consent. The study followed CONSORT guidelines for clinical trials. The study was listed on www.clinicaltrials.gov with registration number (NCT05510882) on 22/08/2022.

### Patient selection

This clinical trial study was conducted on 39 out of 83 patients OSA patients confirmed by polysomnography at sleep disorders breathing Unit, Chest Department at Mansoura University Hospital in the period between July 2022 and July 2023.

### Inclusion and exclusion criteria

**Table:** 

Inclusion criteria	Exclusion criteria
Patient age more than 18 years.	Active infectious disease
OSA patients confirmed by PSG	Previous trauma to the head and neck
	Serious co-morbidity such as chronic or decompensated liver disease, neuromuscular disorders, chronic pulmonary diseases, any cardiac diseases such as rheumatic heart diseases, heart failure, coronary artery disease, myocardial infarction (MI), took up medications that altered pain perception.
	Patients with clinical or radiographic signs of temporomandibular disorders
	Patients refused the procedure.

The patients were randomized 1:1:1 in the 3 groups and the treatments were stratified with age adjustment.

### Sample size calculation

Sample size calculation was based on Apnea hypopnea index between different modalities mandibular advancement device (MAD) group and placebo group [25]. The total sample size was determined to be 13 in each group using G power program version 3.1.9.4 to calculate sample size based on effect size of 1.15, using a 2-tailed test, α error = 0.05 and power = 80.0%.

### Randomization

For this clinical trial with three study groups involving 39 participants (13 per group), a randomized block procedure [26] was used as follows:

1. A block size of 3 was chosen (13 blocks).
2. Possible balanced combinations with one T_1_ (CPAP), one T_2_ (MAD), and one T_3_ (OMT) are calculated as 6 blocks:
  - Block 1: T_1_T_2_T_3_.
  - Block 2: T_1_T_3_T_2_.
  - Block 3: T_2_T_1_T_3_.
  - Block 4: T_2_T_3_T_1_.
  - Block 5: T_3_T_1_T_2_.
  - Block 6: T_3_T_2_T_1_.
3. Blocks were randomly chosen to determine the assignment of all 39 participants. The random sequence was 5, 6, 2, 2, 1, 3, 6, 3, 5, 1, 1, 5, and 3 using Number Sequence Generator (randomnumbergenerator.org). This procedure resulted in 13 participants in each of the three study groups. The random allocation sequence was generated by the statistician. The team enrolled and assigned the participants to interventions.

All participants were undergoing the following:

## Medical and dental history

Focused on night symptoms such as snoring, choking, witnessed apnea, bad dreams, nocturia, and daytime symptoms (morning headache, excessive daytime sleepiness.

## Physical examination

- Included, anthropometric measures: height, weight, body mass index (BMI), epworth sleepiness scale (Arabic version) [27].
- Berlin questioner (Arabic version) [28].

## Dental examination

The patients included were examined by expert and calibrated operators, following the diagnostic criteria for temporomandibular disorders (RDC/ TMDs) [29], the most used and updated TMD diagnostic classification system. Pain scoring was measured through a visual analogue scale (VAS) with 0 scores for no pain and 10 scores for worst pain experienced [30].

- Clicking was evaluated as to its presence = 1 or absence = 0.
- The maximal unassisted, pain-free mouth opening (MIO) was measured in millimeters using a Vernier caliper.
- Lateral movements: were measured as the horizontal distance extending from the maxillary midline to the mandibular midline in millimeters using a Vernier caliper.

Pre-treatment measurement records were considered as a baseline to be used in comparison with 3 months post-treatment records.

## Polysomnography

- Full night attended PSG that will be performed by thoracic medicine technicians and then interpreted by thoracic medicine physicians, under the supervision of “prof. Abdelbaset Saleh “using “SOMNO screen TM plus PSG” with Domino version 2.5.0 “2011-11-24”.
- All patients will undergo full night attended polysomnography in the sleep laboratory of Mansoura University Hospital, Chest department.
- Physiological parameters were recorded during standard PSG to provide adequate data for interpretation [31].
- The polysomnograms were all recorded manually according to the American academy of sleep medicine [32].

## Magnetic resonance imaging (MRI)

All patients have been examined by MRI, and TMJ protocol using Inginea Philips 1.5 tesla. A specific TMJ surface coil was applied. The examination started by an axial scout, then sagittal oblique T1 and proton density (PD) in closed and maximal opening position for both sides were obtained in all patients. Coronal PD images were also obtained for both sides. Parameters used were matrix, field of view (FOV), slices thickness were 256 × 128, 1–12 cm & 3 mm. TR/TE for T1 and PD were 400–500 / 10–14 ms & and 2000 /20 ms respectively.

Both TMJs were assessed using MRI regarding presence or absence of TMD, bone or joint disease before and after the treatment procedure.

### Analysis

- Disc morphology, signal intensity and position were initially evaluated in sagittal oblique PD images, closed position and re assessed in open position.
- Anterior translation of the mandibular condyle and masticatory muscles were evaluated & joint effusion was excluded.
- Sagittal T1 was used for anatomy, and bone evaluation.
- Coronal PD was revised to assess anatomy and exclude medial or lateral disc dislocation.

The patients were divided equally into three treatment groups each including 13 patients.

#### For group I

Patients were treated with CPAP after CPAP titration.

#### For group II

**Fig. 1:**
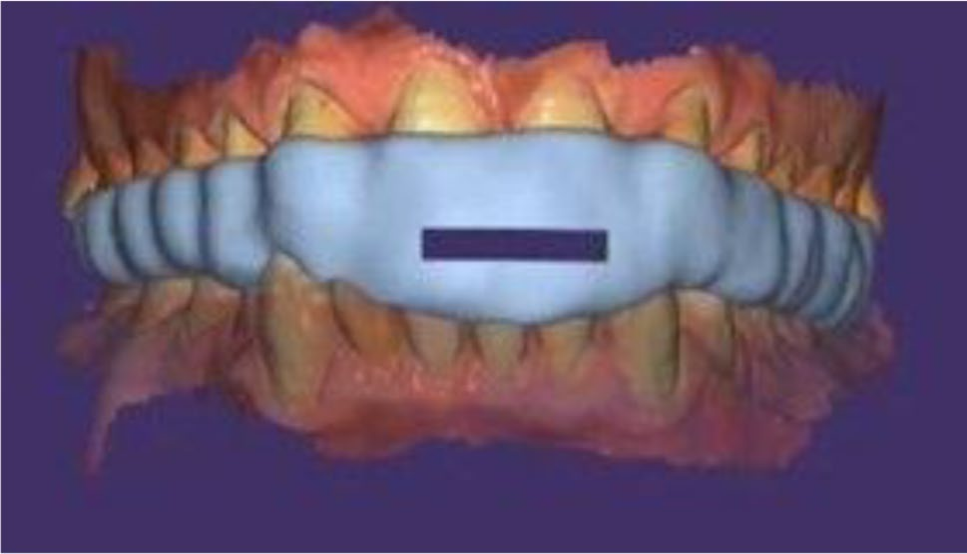
MAD three-dimensional design with opening for breathing designed by exocad software

**Fig. 2:**
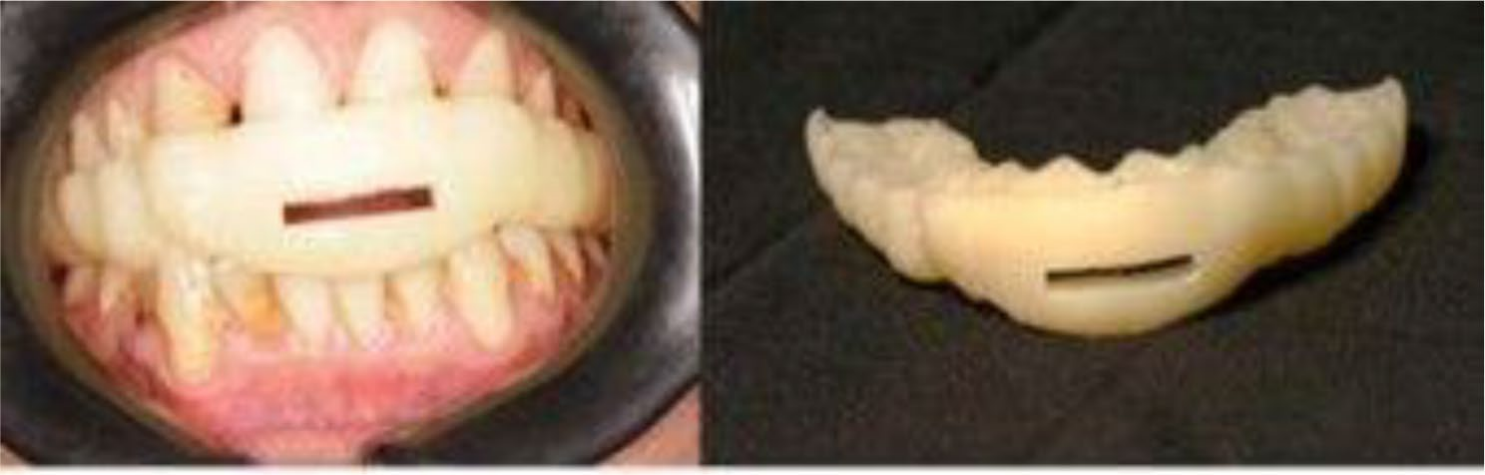
Three-dimensional printed MAD

Patients were treated with digitally fabricated MAD. An intraoral scan of both maxillary and mandibular arches was made. Using leave gauge to help the patient to bite on edge-to-edge position with separation of arches about 2–3 mm between anterior teeth. Bite was recorded with patient biting on the leave gauge using an intraoral scanner by scanning both the right and left sides [33]. After that MAD was constructed on exocad software using splint module (Fig.1). The MAD was designed for both maxillary and mandibular teeth covering the occlusal and incisal third of teeth. Then the designed device was saved as (standardized triangulation language) STL. After that use a digital light processing printer (DLP) 3D printer to print the device (Fig. 2). 1 An FDA−approved resin was used. The device was inserted intraorally, and occlusion was adjusted. For patients with severe OSA, we began with a mandibular protrusion of approximately 70%, and in cases of mild to moderate OSA, we began with that of approximately 50% [34]. Patients were instructed to wear MAD during sleep (Fig. 1).

#### For group III

OMT was the main treatment modality. We were working at increasing the tone and endurance of the targeted muscles of the tongue, soft palate, and pharynx. The therapy was applied twice sessions weekly in a hierarchal manner over three months, the patients were advised to practice the therapy at home regularly at a rate of three to five times for 10 min each per day [35].

A) OMT was either: Non-articulatory OMT: including; 1.tongue stabilization, 2. tongue protrusion out the mouth, 3.tongue lateralization, 4. tongue elevation, 5. holding the tip of the tongue between teeth anteriorly while trying to swallow,6. resistive therapy, and 7. palatal elevation with and without yawn (to feel the soft palatal lift).
B) Articulatory therapy: including (1) through production of Uvular sounds X, Y, and Q (developed by contraction of the uvula) separately several times each, and (2) production of lingo-velar sounds (produced by the dorsum of the tongue and the velum) G, K, separately several times each. The detailed method by Baz et al. [35].

### Statistical analysis

Data were analyzed using IBM-SPSS software (Version 27.0, 2020). Quantitative data were initially tested for normality using Shapiro-Wilk’s test, and z-scores of skewness and kurtosis with data being normally distributed if two of the three supports normality (Shapiro test: *p* > 0.050, z-scores ± 2.58). The boxplots were inspected for the presence of significant outliers. Quantitative data were expressed as mean, standard deviation (SD), and standard error (SE) if normally distributed or median and range if not. The paired-samples t-test was used to compare normally distributed paired data. The one-way ANOVA was used to compare normally distributed data between three groups. One-way ANCOVA was used to compare postintervention data between groups adjusted to preintervention data as a covariate. If assumptions to do one-way ANCOVA were violated, its non-parametric equivalent (Quade’s nonparametric ANCOVA) was used, and results were considered statistically significant if p-value ≤ 0.05 (Fig. 2).

## Results

**Fig. 3:**
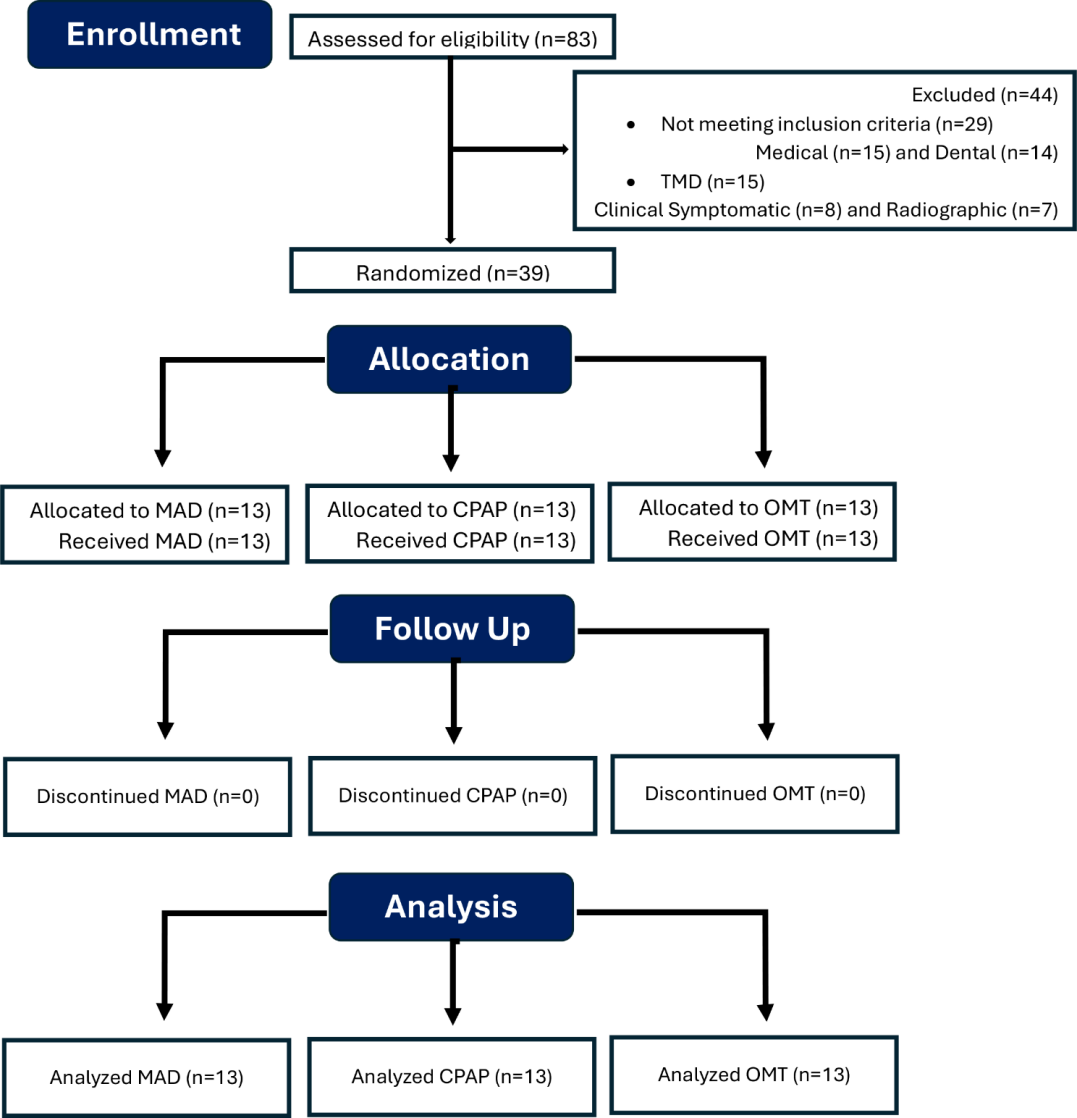
Flow Chart according to the CONSORT guidelines

Out of the 83 patients assessed for eligibility, 44 did not meet inclusion criteria and were excluded and 39 subjects were randomized into three study groups. 13 patients allocated for MAD, 13 patients allocated for CPAP and 13 patients allocated for OMT group; No adverse events were reported during the follow-up period. See the CONSORT Flow Diagram depicted in (Fig. 3) for further details.

The age range was between 19 and 56 years & a mean of (24.51 ± 3.41). Female: Male ratio was 8:5& 6:7&9:4 in the CPAP group & MAD group & OMT group III respectively). The three groups were homogenous regarding age, sex and body mass index.

Regarding the effect of different treatments of sleep apnea on MIO and lateral jaw movements on both sides before the intervention, there was no statistical significance between CPAP, MAD, OMT groups as shown in Table 1.

**Table 1:** MIO and lateral movements in the three studied groups (before the intervention)

Variable	Group						Test of significance	
	CPAP		MAD		OMT			
	Mean	SD	Mean	SD	Mean	SD	F	*p*-value
MIO	39.8	4.3	40.1	5.2	38.1	5.7	0.579	0.566
Movement (right)	7.54	1.76	8.54	1.85	8.23	2.83	0.997	0.384
Movement (left)	7.38	2.33	8.23	1.17	6.69	2.39	2.398	0.115

Notes: Data is mean and standard deviation (SD). The test of significance is one-way ANOVA (standard ANOVA for MIO and Welsh’s ANOVA for movements)

Table 2 showed the results of one-way ANCOVA which was run to compare the 3 months after the intervention MIO and lateral movements (right and left) adjusted to the values before the intervention. There was no statistically significant difference between the three groups as regards all three parameters.

**Table 2:** MIO and movements in the three groups (after the intervention)

Variable/Group	Unadjusted		Adjusted		Test of significance		
	Mean	SD	Mean	SE	F	*p*-value	η_*p*_^2^
MIO					2.215	0.112	0.112
CPAP	40.62	4.89	40.34	0.608			
MAD	40.23	4.97	39.67	0.610			
OMT	37.69	5.09	38.53	0.612			
Movement (right)					2.665`	0.084	0.132
CPAP	7.85	1.57	8.26	0.201			
MAD	8.62	1.12	8.50	0.197			
OMT	8.15	1.52	7.89	0.199			
Movement (left)					1.309	0.283	0.070
CPAP	7.77	3.03	7.82	0.411			
MAD	9.00	1.96	8.24	0.422			
OMT	6.54	2.22	7.25	0.421			

Notes: Data is mean & standard deviation (SD) for unadjusted values after the intervention and mean & standard error (SE) for values after the intervention adjusted to the values before the intervention as a covariate. The test of significance is one-way ANCOVA

η_p_^2^ stands for partial eta squared

In Table 3 there was a statistically significantly higher score after the intervention vs. score before the intervention in OMT group regarding MIO with a medium effect size (Cohen’s d = 0.629). There was no statistically significant difference for all other parameters.

**Table 3:** Comparisons in each group before vs. after the intervention

Variable / Group	Before the intervention			After the intervention			Test of significance		Effect size
	Mean	SD	95% CI	Mean	SD	95% CI	t (12)	*p*-value	
MIO									
CPAP	39.77	4.32	37.16–42.38	40.62	4.89	37.66–43.57	-0.935	0.368	0.259 (0.251)
MAD	40.08	5.24	36.91–43.24	40.23	4.97	37.23–43.23	-0.379	0.711	0.105 (0.102)
OMT	38.54	5.17	35.41–41.67	37.69	5.09	34.62–40.77	2.269	**0.043**	0.629 (0.609)
**Movement (right)**									
CPAP	7.54	1.76	6.47–8.60	7.85	1.57	6.90–8.80	-1.477	0.165	0.410 (0.397)
MAD	8.23	1.36	7.41–9.05	8.62	1.12	7.94–9.29	-1.443	0.175	0.400 (0.388)
OMT	8.46	1.66	7.46–9.47	8.15	1.52	7.24–9.07	1.760	0.104	0.488 (0.473)
**Movement (left)**									
CPAP	7.38	2.33	5.98–8.79	7.77	3.03	5.94–9.60	-0.862	0.406	0.239 (0.231)
MAD	8.23	1.17	7.53–8.94	9.00	1.96	7.82–10.18	-1.552	0.147	0.431 (0.417)
OMT	6.69	2.39	5.25–8.14	6.54	2.22	5.20–7.88	0.693	0.502	0.192 (0.186)

Notes: Data is mean and standard deviation (SD). The test of significance is the paired-samples t-test. effect size is Cohen’s d (Hedges’ correction). Effect size is small, medium, and large if it 0.2, 0.5, and 0.8, respectively. 95% CI = 95% confidence interval

Table 4 showed that the pain score was statistically significantly higher at 3 months after the intervention (*p* = 0.001) in OMT vs. the two other groups.

**Table 4:** Pain score in the three groups

Group	Pain score						p-value*
	before the intervention			3months after the intervention			
	Median	Minimum	Maximum	Median	Minimum	Maximum	
CPAP	0	0	0	0	0	0	**0.001**
MAD	0	0	0	0	0	0	
OMT	0	0	1	1	0	3	

Notes: *p-value by using Quade’s nonparametric ANCOVA

### Results of MRI

**Fig. 4:**
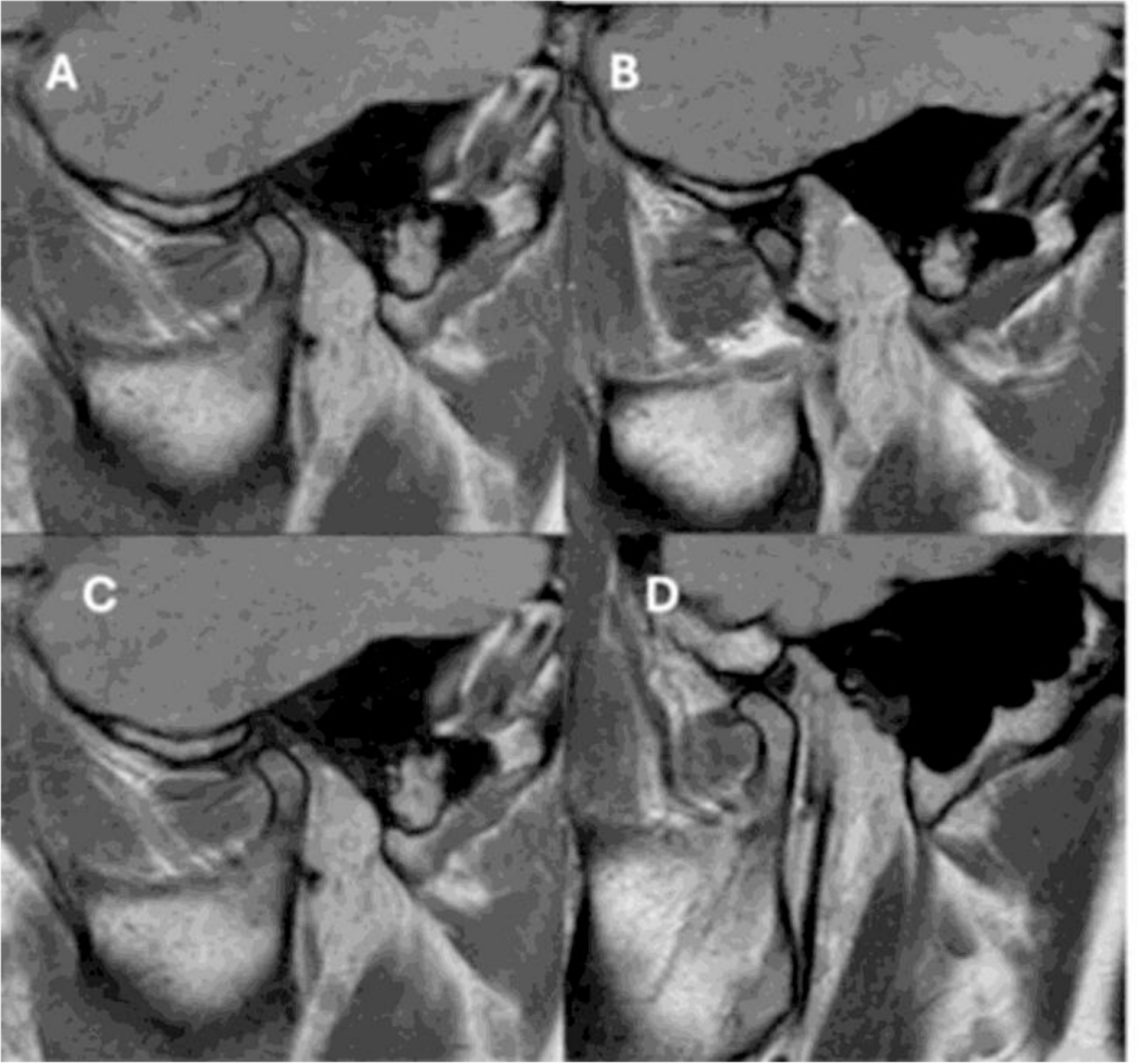
**(A** & **B**) before the intervention, **C&D** after the intervention in OMT group, show normal unchanged disc shape and position in closed and open position

There was no significant difference between before and after the intervention follow-up period in the three studied groups. (Fig. 4)

## Discussion

This is the first clinical trial study in which the effect of different treatment modalities (CPAP, MAD, and OMT) of OSA patients has been evaluated on TMJ health.

The CPAP, MAD, and OMT were approved in other literature to reduce AHI and daytime sleepiness and improve the quality of sleep in patients affected by OSA [12, 36–38]. So, in the current study we only evaluate the effect of those treatments on the health of TMJ despite their effect in the management of OSA.

Patients suffering OSA signs and symptoms are more likely to present the first onset TMD [17], The link between TMD pain and sleep fragmentation is still not well understood, but it seems to have a reciprocal influence [39]. When deciding on the most appropriate treatment option for obstructive sleep apnea, it is important to consider individual patient factors and preferences [40], considering the potential effects of the selected treatment on the patient’s bite, the health of the TMJ, the patient’s quality of life, and work performance [41, 42].

The RDC/ TMDs international protocol was used to evaluate the prevalence of TMDs in a group of adult patients who were diagnosed with OSA after PSG. MRI evaluation for the TMJ was performed for the three groups before the beginning of the treatment and after 3 months of follow-up to confirm the presence or absence of temporomandibular disorders. There were no significant changes found in the MRI in the three studied groups before and after the intervention, all the groups showed normal unchanged disc shape and position in closed and open position.

The results highlighted no significant differences between the CPAP, MAD, and OMT regarding lateral movements and clicking. Knappe et al. [43], found significant changes in clicking in the short-term follow-up (3 months) when used MAD in the treatment of OSA. This was in disagreement with our study, and this may be due to using digitally fabricated MAD with separation of only 2–3 mm and mandibular protrusion edge to edge that didn’t affect the disc condyle relationship.

It has been reported that TMD pain causes a decrease in quality of life [44], and it was important to measure the intensity of pain in relation to CPAP, MAD, and OMT. TMD pain intensity was measured using pain scoring of visual analogue scale (VAS) with 0 scores for no pain and 10 scores for worst pain experienced [25].

The linkage between TMD pain and OSA may be found in the hyperalgesia that OSA patients might experience, in relation to the fragmented sleep and hypoxemia that might enhance peripheral and central pain sensitization, thus increasing spontaneous pain [45, 46, 47].

There was a significant difference in pain score and MIO measurements in the OMT group as compared to CPAP and MAD. The pain score in OMT after 3 months of follow-up was mild pain (maximum score 3) and the MIO measurement was still in the normal range of 38.54 ± 5.17 [48].

We presume that, this can be attributed to the strain in the muscles of the temporomandibular complex or the capsular ligament of the temporomandibular joint in the OMT; thus, the patient practiced the therapy regularly at home, three to five times per day with minimum 10 min for each time. There was no TMJ articular change in the MRI after the 3-month follow-up period in the OMT group.

These results were in accordance with Bartolucci et al. [49] recent cross-sectional study that showed, the prevalence of TMDs in adults with OSA was not significantly higher compared to healthy controls in their cohort [49].

M Nikolopoulou et al., [25] found no significant difference between using CPAP and MAD regarding pain, lateral movement, and MIO which was in accordance with our results.

To the best of our knowledge, this is the first study assessing the impact of different treatments of OSA (CPAP, MAD, and OMT) on TMJ signs and symptoms (pain, clicking, MIO, and lateral movements). Yet, this study presents some limitations: as long-term follow-up and a larger sample size are required.

We recommend: future studies with longer follow -up periods, and further studies about the combination of orofacial myofunctional therapy and myofascial release in the management of OSA patients to avoid the appearance of muscle fatigue that leads to mild pain and slight limitation of MIO.

## Conclusion

CPAP and MAD are better for preserving the health of TMJ in the treatment of OSA patients. While OMT showed mild pain and slight limitation of MIO (that is still within the normal range of mouth opening) compared to CPAP and MAD.

### Electronic supplementary material

Below is the link to the electronic supplementary material.

Supplementary Material 1

## Data Availability

The data sets used and/or analyzed during the current study are available from the corresponding author on reasonable request.

## Footnotes

1 shining3d- accufab-l4k.

## Abbreviations

OSA: Obstructive sleep apnea
TMJ: Tmporomandibular joint
CPAP: Continuous positive airway pressure
MAD: Mandibular advancement device
OMT: Oral myofunctional therapy
MRI: Magnetic resonance imaging
AHI: apnea-hypopnea index
PSG: Polysomnography
TMD: temporomandibular disorder
VAS: Visual analogue scale
MIO: Maximum interincisal opening
TMD: Temporomandibular disorder
PD: Proton density
RDC/ TMDs: Research Diagnostic Criteria for Temporomandibular Disorders
